# A Case Report Illustrating the Postoperative Course of Descemetorhexis without Endothelial Keratoplasty with Topical Netarsudil Therapy

**DOI:** 10.1155/2019/6139026

**Published:** 2019-10-13

**Authors:** Pimpiroon Ploysangam, Sangita P. Patel

**Affiliations:** ^1^Ross Eye Institute, Department of Ophthalmology, Jacobs School of Medicine and Biomedical Sciences, State University of New York at Buffalo, Buffalo, NY, USA; ^2^Research and Ophthalmology Services, Veterans Administration of Western New York Healthcare System, Buffalo, NY, USA

## Abstract

Fuchs endothelial corneal dystrophy (FECD) is the most common indication for corneal transplantation in the United States. Recently, descemetorhexis without endothelial keratoplasty (DWEK) or Descemet's stripping only (DSO) has become an attractive alternative to corneal transplantation for these patients. DSO circumvents the challenges associated with cadaveric donor corneal transplantation by tapping into the potential of the patient's own corneal endothelium to repair defects. Outcomes have been variable with emerging knowledge on predictive factors for success. Our case describes a 51-year-old patient with visually significant confluent central guttae from FECD who underwent a successful DSO with immediate post-operative use of the Rho-associated protein kinase (ROCK) inhibitor (netarsudil). We report the preoperative and post-operative slit lamp images, specular microscopy data, and corneal topography, thickness, and densitometry data. These represent a unique data set for this new surgical treatment option for FECD. Despite a small descemetorhexis, we show the improvement in corneal thickness and opacity extends beyond the boundaries of the descemetorhexis. Early initiation of a ROCK inhibitor was a successful treatment for this patient.

## 1. Introduction

Fuchs endothelial corneal dystrophy (FECD) is the most common endothelial dystrophy, with its pathology characterized by the presence of deposits (guttae) in the basement membrane (Descemet's membrane) of the corneal endothelium and decreased endothelial cell density [1]. Treatment for advanced FECD is by corneal transplantation, and FECD accounts for more than 17,000 corneal transplants in the United States annually [2]. All of these surgical interventions for FECD require cadaveric donor tissue and carry the associated risks of graft rejection and failure. Transplantation options include penetrating keratoplasty (PKP), Descemet stripping endothelial keratoplasty (DSEK), and Descemet membrane endothelial keratoplasty (DMEK) [3, 4]. Cell therapy with Rho kinase (ROCK) inhibitors and cultured donor corneal endothelial cells has also recently been described [5]. Rho kinase halts progression of corneal endothelial cells through the cell cycle; inhibition leads to proliferation, attachment to substrate, and suppression of apoptosis in corneal endothelial cells [6].

Although it was thought that corneal endothelial cells were arrested in the cell cycle, recent cases show that descemetorhexis with removal of the diseased Descemet's membrane and endothelium can promote cell repopulation [7, 8]. The guttae microenvironment itself affects endothelial cell behavior. Large guttae stimulate apoptosis of corneal endothelial cells and increase markers of cell stress [9]. Descemet's stripping only (DSO), also known as Descemetorhexis without endothelial keratoplasty (DWEK), has thus emerged as a new alterative to corneal transplantation. DSO involves removing the central area of diseased Descemet's membrane and corneal endothelial cells via a descemetorhexis in order to allow peripheral corneal endothelial cells to repopulate the central corneal endothelial monolayer. DSO has the benefit of bypassing a need for donor tissue altogether, removing the risks associated with graft rejection and failure. Much is still unknown regarding this procedure including factors that would predict success [10]. While there are multiple case series on DSO, our case report details the pre- and postoperative course and associated clinical imaging of a patient with FECD undergoing DSO. This patient was also placed on ROCK inhibitor therapy immediately postoperatively, which currently has only been used in patients with nonclearing corneas after DSO [11].

## 2. Case Presentation

Our patient is a 51-year-old Caucasian female with history of FECD who presented with gradually worsening vision over the past year. She had noted fluctuating vision, photophobia, difficulty reading, and glare with oncoming headlights while driving. Her ocular history was remarkable for ocular rosacea with inspissated meibum and eyelid margin vascularization and glaucoma suspect due to cup to disc asymmetry. Ocular medications included intermittent use of artificial tears and sodium chloride 5% ointment.

**Figure 1:**
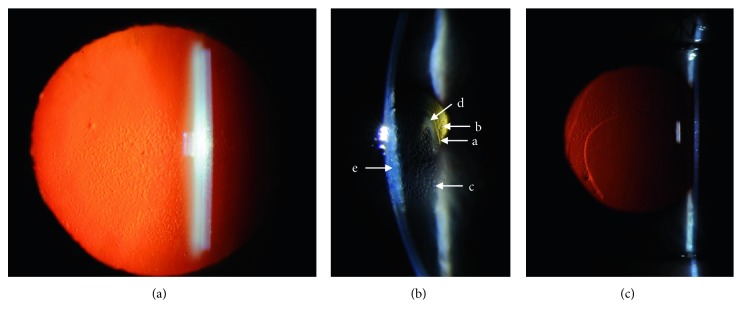
Slit lamp biomicroscopy photos pre- and post-DSO surgery for the left eye. (a) Preoperative red reflex photo with a dilated pupil shows confluent guttae in the visual axis, slightly greater in the inferior half of the visual axis compared to superior half. The peripheral cornea is relatively spared of guttae. (b) Slit photo through the central cornea at 1 week post-DSO surgery demonstrates a. the edge of the descemetorhexis; b. guttae outside the area of Descemet's stripping; c. reticular epithelial edema; d. peripheral ring of corneal clearing without edema in presumed area of endothelial healing; and e. corneal stromal edema. (c) One month postoperative red reflex photo shows inferocentral descemetorhexis with clear visual axis without guttae and peripheral nonconfluent guttae.

**Figure 2:**
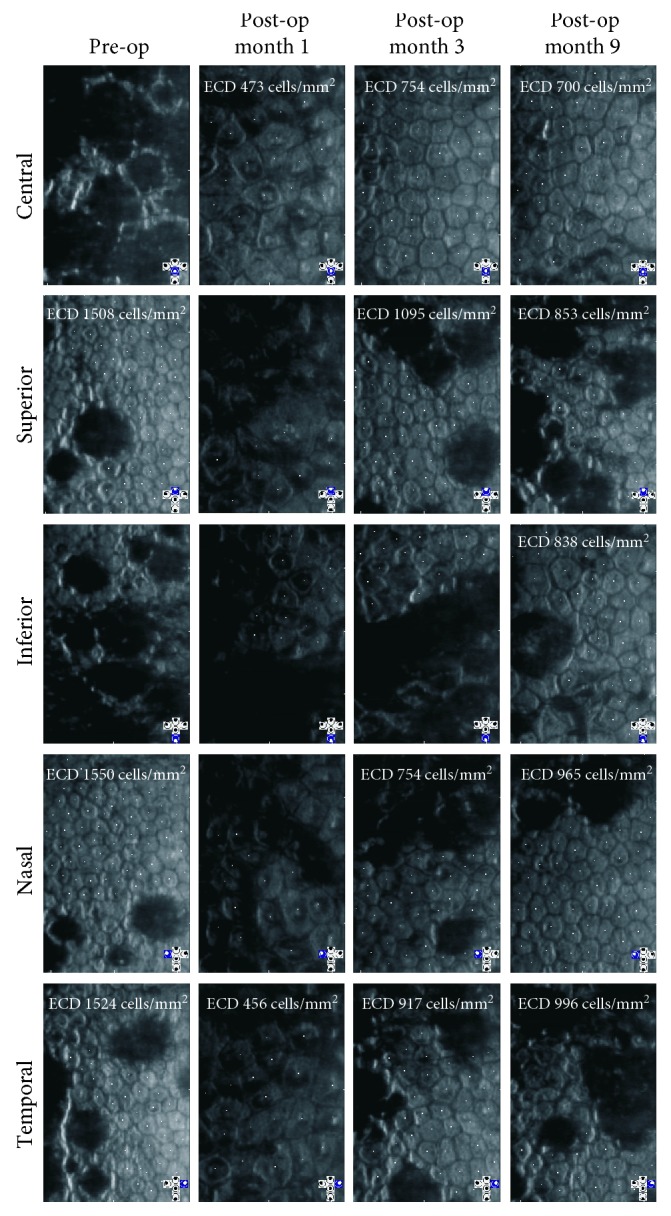
Specular microscopy imaging of the corneal endothelium pre- and post-DSO surgery for the left eye. The locations (central, superior, inferior, nasal, and temporal) are based upon the standard gaze positions of the Konan specular microscope. Endothelial cell density (ECD) is indicated when measurable.

**Figure 3:**
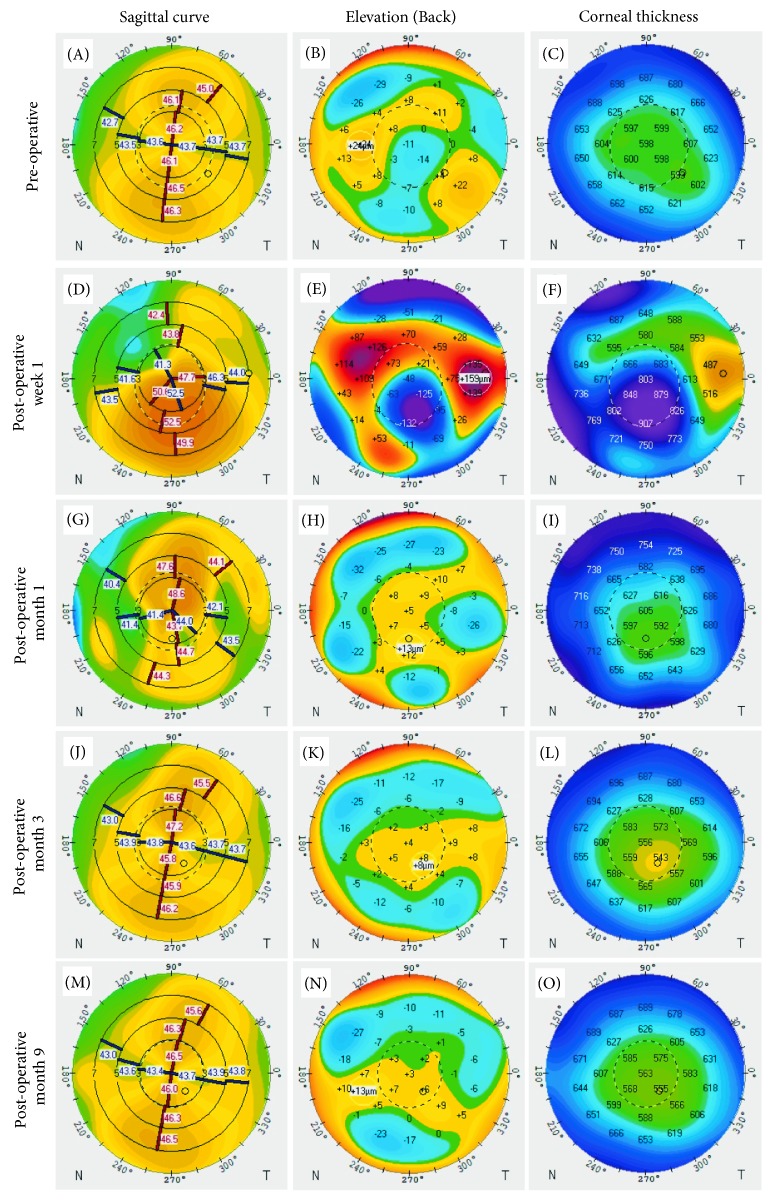
Sagittal corneal curvature, back surface elevation, and pachymetry maps pre- and post-DSO for the left eye.

Her eye exam revealed a corrected Snellen visual acuity of 20/40 (right; −0.75 +3.00 × 100) and 20/30 (left; −0.75 +2.75 × 085). Her corneas had central confluent endothelial guttae in both eyes measuring 4.0 × 4.0 mm (right) and 3.8 × 4.0 mm (left) with associated stromal haze in both eyes (Figure 1(a)). The peripheral corneas in both eyes were relatively spared of guttae. There was no clinically apparent corneal edema on slit lamp exam. Ultrasound pachymetry (DGH 555 Pachette 3 Ultrasonic Pachymeter, DGH Technology, Inc., Exton, PA) showed a central corneal thickness of 608 *µ*m (right) and 595 *µ*m (left). She also had 1+ nuclear sclerotic cataract in both eyes. Dilated fundus exam was unremarkable. Specular microscopy imaging (Konan Specular Microscope Noncon Robo Series NSP-9900, Konan Medical Inc., Japan) was completed and showed central confluent guttae (unable to count endothelial cell density). The peripheral cornea showed scattered, nonconfluent guttae with cell densities of 1508–1550 cells/mm^2^ in the left eye (Figure 2). The location of “peripheral” corneal endothelial imaging by the Konan Specular Microscope is not specified, but we suspect a midperipheral location based upon the distribution of guttae in the corresponding red-reflex image (Figure 1(a)). Scheimpflug tomography (Pentacam, Oculus, Inc., Arlington, WA) showed 2 diopters of regular with-the-rule astigmatism (Figure 3(a)). The minimum central thickness was 593 *µ*m in the left eye and the decreased back surface elevation suggested the presence of subclinical corneal edema (Figure 3(b)).

The patient's visual symptoms were felt to be secondary to corneal changes from FECD and mild cataract. Surgical options were reviewed with her including cataract extraction with intraocular lens placement with or without DSEK or DSO. Patient decided to proceed with cataract extraction with intraocular lens placement and DSO for her left eye, with the understanding that she might need corneal transplant should her cornea fail to clear following DSO.

The patient underwent routine cataract surgery with phacoemulsification and intraocular lens placement under topical anesthesia. Following lens implantation, the central corneal area of densest guttae was marked at 4 × 4 mm with calipers on the corneal surface. While the eye was still filled with cohesive viscoelastic, a reverse sinskey hook was used to score and lift the Descemet's membrane/endothelium for approximately 1–2 clock hours in the marked region. Using the lifted edge of Descemet's membrane, a continuous descemetorhexis was pulled approximating the marked central region using utrata forceps. Viscoelastic was then removed from the anterior chamber, and the remainder of the case was completed per standard cataract surgery protocol.

On her postoperative day 1 visit, uncorrected vision in the left eye was 20/125 (pinhole to 20/60). Her cornea showed removal of greatest area of guttae in her left eye measuring 4 mm (vertical) × 3.4 mm (horizontal), with slight inferior displacement of area of stripping relative to the pupil, corresponding to the area with densest guttae preoperatively (Figure 1(a)). Ultrasound pachymetry was 936 *µ*m. The anterior chamber was quiet, and posterior chamber intraocular lens was in proper position. The patient was started on the following eye drop regimen for the left eye: prednisolone acetate 1% 4x/day, polymyxin b sulfate-trimethoprim 4x/day, netarsudil at bedtime, and polymyxin B/neomycin/dexamethasone ointment at bedtime.

At postoperative week 1, her visual acuity in the left eye was 20/250 (pinhole to 20/100). Her cornea showed stromal and focal reticular epithelial edema in the left eye measuring 3 mm (vertical) × 2.8 mm (horizontal). Reticular edema has previously been noted with netarsudil use [12]. The area of stripping was clearly delineated and edges of this area were noted to be clearing (Figure 1(b)). The anterior chamber remained deep and quiet. Scheimpflug tomography imaging (Pentacam) showed inferocentral thickening and steepening in the area of Descemet's stripping (Figure 3(d)–3(f)). At this visit, the patient noted pain which was thought to be secondary to the corneal epithelial edema. Frequent application of artificial tears did not alleviate the pain. A bandage soft contact lens was placed for 1 week. All eye medications were continued except for the polymyxin B / neomycin/dexamethasone ointment.

At the postoperative one-month visit, her uncorrected vision measured 20/150 (pinhole 20/50). The corneal edema had resolved, but she continued to have a central anterior stromal haze. Scheimpflug tomography (Pentacam) showed a minimum central thickness at 565 *µ*m (Figure 3(i)). The endothelium could be imaged by specular microscopy (Konan) and showed central endothelial cell counts of approximately 473 cells/mm^2^. The polymyxin B sulfate-trimethoprim and netarsudil eye drops were stopped, and the prednisolone acetate 1% eye drop was tapered off over the next 3 weeks.

Her vision continued to improve at postoperative month two, with her best corrected vision at 20/40 (right; −0.75 +3.00 × 100 OD) and 20/25 (left; −1.50 +2.25 × 080). Due to persistent pain, a bandage soft contact lens was again placed at the postoperative month two visit for one more week, and the patient was started on fluorometholone eye drops 2x/day in the left eye. Pain has been reported as a side effect of instillation of netarsudil eye drops; however, she had stopped using netarsudil eye drops 1 month prior [13].

**Figure 4:**
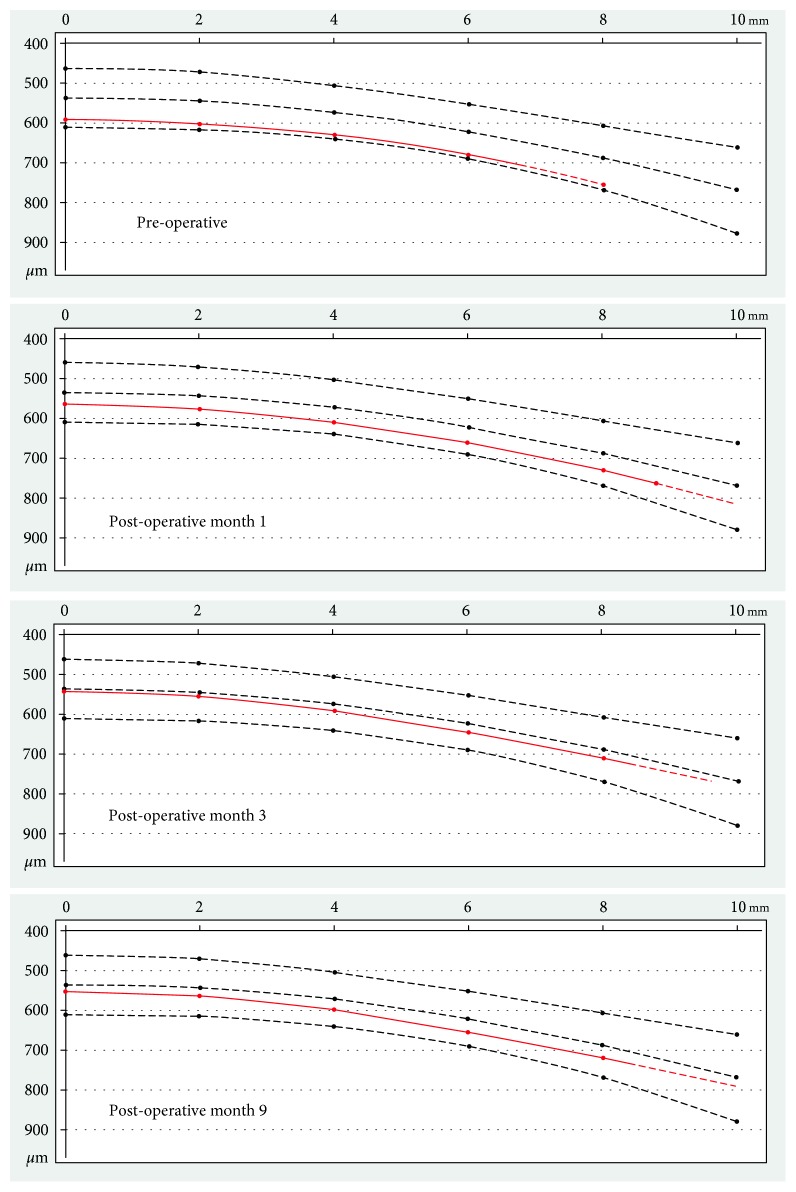
Corneal thickness spatial profile pre-and post-DSO for the left eye. The corneal thickness decrease following DSO is not limited to the central area of stripping but also extends to the peripheral cornea.

**Figure 5:**
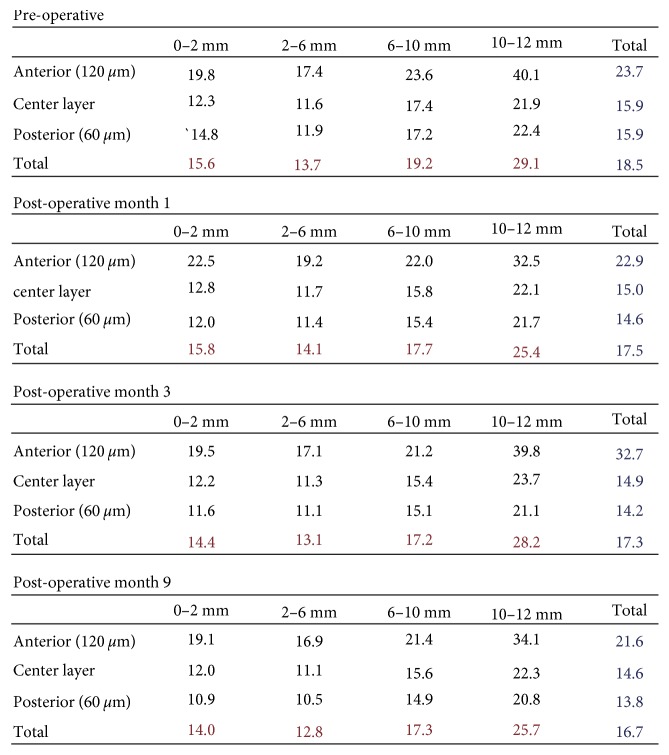
Corneal densitometries pre- and post-DSO for the left eye. Decrease in densitometry is noted not only in the posterior layer centrally (the region of Descemet's stripping), but also in the posterior layer peripherally and in the anterior layer throughout.

At 3 months, vision remained stable from the prior month. Specular microscopy (Konan) showed a central cell density of 754 cells/mm^2^ (Figure 2). Scheimpflug tomography (Pentacam) demonstrated decreased corneal thickness compared to all prior measurements for the left eye (Figures 3(l) and 4). In addition, evaluation of corneal opacity by Scheimpflug corneal densitometry showed improved (reduced) densitometry values from both the anterior and posterior corneal layers, and from the central and peripheral corneal zones compared to preoperatively (Figure 5). She continued to have pain in her left eye which did not respond to bandage contact lens placement but was alleviated with periodic use of fluorometholone 0.1% eye drops once daily as needed. The etiology of the pain was not evident on exam, thus we suspect a neuropathic component. Fluoromethalone eye drops were continued for an additional 2 months. At 9 months postoperatively, her left eye pain had resolved. Her central corneal endothelial cell density was 700 cells/mm^2^ and corneal pachymetry was 555 *µ*m (Figures 2 and 3). The cell density of the peripheral corneal endothelium was noted to be decreased from pre-operatively.

## 3. Discussion

Our patient had a successful outcome from DSO surgery with improved corneal thickness, densitometry, and visual acuity, and rapidly repopulated central corneal endothelium without guttae. The cell density of the central corneal endothelium could be measured by specular microscopy at 1 month after DSO surgery indicating healing of the endothelial monolayer and sufficient endothelial function to improve corneal edema to allow for imaging by that time point. Her visual acuity likely improved as a result of both cataract extraction and DSO. Although no clear predictive factors for success have been established for descemetorhexis [10], Moloney et al. have reported criteria for patient selection based on their successful pilot study; this includes visual symptoms from central guttae and not corneal edema, presence of healthy peripheral endothelial cell population, and symptoms disruptive enough to qualify for a graft otherwise [11]. Our patient met these criteria. Successful outcomes of DSO have also been attributed to surgical technique, with continuous curvilinear descemetorhexis having better outcomes than descemetorhexis with 360° scoring [10]. For this patient, we used the successful continuous curvilinear descemetorhexis technique.

ROCK inhibitors have been used off-label for DSO surgery to promote corneal endothelial proliferation and clearing of the cornea when the corneal edema fails to resolve. Moloney et al. performed a series of successful DSO cases and used ROCK inhibitors in only the few cases that failed [11]. Two failed cases received the topical ROCK inhibitor ripasudil six times daily for two weeks, with subsequent complete resolution of cornea edema. In one failed case with a detached Descemet's membrane, topical compounded ROCK inhibitor Y-27632 failed to improved edema, and eventually the patient required endothelial keratoplasty [11]. The enhanced surgical success with ROCK inhibitor opens the possibility of extending descemetorhexis to previously borderline candidates [10]. Notably, our case describes one of the first to supplement DSO with netarsudil, and our post-operative course is similar to cases supplemented with other ROCK inhibitors, specifically ripasudil. Based upon our case report, we speculate that netarsudil can replicate the accelerated recovery noted with other ROCK inhibitors; however, we cannot verify this without a well-designed clinical trial.

*In vitro* studies on corneal endothelial wound healing with ROCK inhibitors show that cell cycle progression (i.e. corneal endothelial cell proliferation) is greatest closest to the wound edge and stops as soon as the wound is healed [14, 15]. These data suggest that the greatest benefit for enhancing corneal endothelial cell proliferation and future cell density would be immediately after surgery when the endothelial wound is open, rather than waiting later in the healing course. We therefore started our patient on a ROCK inhibitor (netarsudil) immediately following surgery. We selected netarsudil due to its commercial availability in the United States, its pharmacologic profile showing similar potency to Y-27632 for block of rho-kinase isoforms present in corneal endothelium [16] and its use in prior human clinical studies [17]. Given that results with DSOs have been highly variable and little is currently known regarding predictive factors for success, early initiation of ROCK inhibitor may help promote successful outcomes. In our patient, we noticed that the peripheral endothelial cell density decreased following DSO as the central cell density increased. This suggests that while netarsudil may be increasing corneal endothelial cell proliferation, repopulation of the endothelial monolayer likely also involves migration of cells from the peripheral cornea.

Interestingly, although the area of descemetorhexis for this patient was small, we noted improvement throughout the cornea. As expected from removal of guttae by descemetorhexis, corneal densitometry values decreased in the posterior corneal layer in the area of the descemetorhexis postoperatively. However, there was also a diffuse decrease in values across the whole cornea postoperatively, including in the anterior layers of the cornea and in the peripheral cornea outside the area of the descemetorhexis. Likewise, corneal pachymetry measurements decreased not only in the central area of stripping, but also in the peripheral cornea. These findings suggest that even a very small area of diseased Descemet's membrane removed during DSO surgery can have significant impact on the whole cornea.

## 4. Conclusion

Our study and published literature support DSO as a viable alternative to cadaveric corneal transplantation for a select group of FECD patients. The use of ROCK inhibitors may further extend this option to a larger group of patients. Additionally, based upon our patient data, a small descemetorhexis positively impacts corneal thickness and densitometry globally. This may prove helpful as a smaller descemetorhexis leaves a smaller area for healing, which may reduce the time for healing. Although our patient's outcome is promising, long-term outcomes data are still lacking for this procedure with or without ROCK inhibitors.
